# *Erratum*: Vol. 68, No. SS-11

**DOI:** 10.15585/mmwr.mm6948a12

**Published:** 2020-12-04

In the Surveillance Summary “Abortion Surveillance — United States, 2016,” data on the number of known previous induced abortions for women having abortions in this reporting year were erroneously included for New York City. These estimates did not meet reporting standards and should have been excluded from this report. When corrected, among women with abortions in the reporting year, the proportion with no previous induced abortions increased, and the proportion with one or more previous induced abortions decreased.

On page 2, the second sentence of the first paragraph should have read “Women with one or more previous induced abortions accounted for **40.7%** of abortions, and women with no previous abortions accounted for **59.4%**.”

On page 10, the first paragraph of the first column should have read “Data from the **41** areas that reported the number of previous abortions for women who obtained abortions in 2016 indicate that the majority (**59.4%**) had no previous abortions, **34.4%** had one or two previous abortions, and **6.3%** had three or more previous abortions (Table 17). Among the **34** reporting areas***** that provided data for the relevant years of comparison (2007 versus 2016, 2007 versus 2011, 2012 versus 2016, and 2015 versus 2016), the percentage of women who had no previous abortions increased **3%** (from **57.4% to 59.1%**), whereas a **4%** decrease occurred among women who had one or two previous abortions, and a 4% **decrease** occurred among women who had three or more previous abortions from 2007 to 2016. However, the percentage of women who had no previous abortions decreased **1%** from 2007 to 2011 (from **57.4% to 56.8%**) and then increased 3% from 2012 to 2016 (from **57.6% to 59.1%**). By contrast, the percentage of women who had three or more previous abortions increased **4%** from 2007 to 2011 (from **6.8% to 7.1%**) then decreased **6%** from 2012 to 2016 (from **6.9% to 6.5%**). The percentage of women who had one or two previous abortions increased **1%** from 2007 to 2011 (**35.8% to 36.1%**) and then decreased **3%** from 2012 to 2016 (from **35.5% to 34.5%**).” New York City should have been included in the ***** footnote, which lists reporting areas that were not included in these estimates.

In Table 17, the line for New York City should be deleted. For the total line, the numbers and percentages should have read **244,362 (59.4), 100,248 (24.4), 40,972 (10.0), 25,986 (6.3), 411,568 (98.5)**. The * footnote should have read “Data from **41** reporting areas; excludes **11** areas (California, District of Columbia, Florida, Illinois, Maryland, New Hampshire, New Mexico, **New York City**, New York State, Wisconsin, and Wyoming) that did not report, did not report by the number of previous induced abortions, or did not meet reporting standards.” The total in the ** footnote should have been **417,809**.

